# Unexpected Sudden Deaths Following the Co-administration of Ceftriaxone and Lansoprazole: A Case Series

**DOI:** 10.7759/cureus.64143

**Published:** 2024-07-09

**Authors:** Taku Harada, Toshiyuki Nakanishi, Satoshi Kutsuna, Mori Nakai

**Affiliations:** 1 General Medicine, Nerima Hikarigaoka Hospital, Tokyo, JPN; 2 Diagnostic and Generalist Medicine, Dokkyo Medical University Hospital, Mibu, JPN; 3 Medicine, Osaka University, Osaka, JPN

**Keywords:** drug-drug interactions, cardiac sudden death, lansoprazole, qt prolongation, ceftriaxone

## Abstract

Ceftriaxone and lansoprazole are commonly used in clinical settings, but recent analyses indicate a potential risk for QTc prolongation and cardiac events when used together. This case series examines three patients from a cohort of sudden death cases at a single institution over a decade, who received both medications within 24 hours before death. Three cases were identified, each with contributing factors for cardiac arrhythmias. The results underscore the importance of monitoring and possibly avoiding this drug combination in patients at risk of QT prolongation, pending further investigation into the underlying mechanisms.

## Introduction

Ceftriaxone is a third-generation cephalosporin antibiotic widely used in various settings, primarily for pneumonia and urinary tract infections [1-3]. Lansoprazole, on the other hand, is a proton pump inhibitor (PPI) frequently used in routine clinical practice [4-6]. According to a systematic review, lansoprazole accounts for approximately 9% of PPI use across 23 countries [7]. Therefore, it is common for patients already taking lansoprazole to acquire an acute infection and be treated with ceftriaxone [6,8].

A recent large-scale data analysis in the United States has suggested the possibility of a 0.12 ms prolongation of the QTc interval exclusively when lansoprazole and ceftriaxone are used together. The QT prolongation effect is not observed with other PPIs or third-generation cephalosporins and is presumed to manifest exclusively when the aforementioned drugs are combined. The precise mechanism underlying this phenomenon remains elusive but is speculated to involve the blockade of the human ether-a-go-go-related gene (hERG) channel [9]. The hERG channel is present in cardiac cells and regulates the electrical activity of the heart by expelling potassium ions (K⁺). Lansoprazole can inhibit the function of the hERG channel. When ceftriaxone and lansoprazole are used together, lansoprazole binds to the hERG channel, enhancing its inhibitory effect. Lorberbaum et al. conducted electrophysiological tests and confirmed the prolongation of the QT interval, but the molecular mechanism remains unclear [9]. Potential mechanisms might involve a chemical interaction between the two compounds, cooperative binding to the channel, or an indirect effect mediated by proteins associated with hERG [9]. Based on this hypothesis, a retrospective observational study was conducted in 13 Canadian hospitals involving 31,152 patients administered ceftriaxone, and it was reported that the lansoprazole group had a significantly higher frequency of ventricular arrhythmias or cardiac arrest compared to the group taking other PPIs (3.4% vs. 1.2%) [6]. In a study using a large-scale database in Japan, Mitsuboshi et al. reported that the concomitant use of ceftriaxone and lansoprazole increases the risk of ventricular arrhythmias and cardiac arrest [8]. However, this large-scale retrospective observational study has limitations, such as not being able to identify on which day adverse events occurred after ceftriaxone administration, lack of electrocardiogram (ECG) data, and a lack of verification of hypomagnesemia, which could cause QT prolongation. The hypothesized relationship between QT prolongation, sudden death, and the combined use of ceftriaxone and lansoprazole has only just begun to be examined. However, given the frequency of use of the relevant drugs and the severity of outcomes, further detailed investigation is deemed necessary. This case series was conducted to clarify the characteristics of cases of sudden death due to the combined use of ceftriaxone and lansoprazole, including ECG examinations at the time of hospitalization and the timing of occurrence.

## Case presentation

Methods

From October 2013 to September 2023, patients who were hospitalized in the Department of General Medicine at Nerima Hikarigaoka Hospital and died in hospital were evaluated. As there are no established definitions for cardiac arrest or unexpected in-hospital sudden death [10,11], sudden death was defined in this study as meeting all of the following six criteria: no circulatory failure (blood pressure maintained without vasopressors) or respiratory failure (oxygen supplementation satisfied with ≤4 L/min) at 6 h before death; no exacerbating acute illnesses; no witnessed choking, aspiration, or airway obstruction; not in the terminal stage of malignancy or chronic diseases; not under palliative care; and first found in a state of fatal arrhythmia (ventricular fibrillation {VF}, pulseless ventricular tachycardia {VT}, or torsade de pointes) or cardiac arrest. From among the cases that experienced sudden death, those who had been administered ceftriaxone and lansoprazole within 24 h before death were selected, and the following information was collected: age, sex, underlying diseases, admission illness, ECG findings at admission, main clinical course before death, risk factors for ventricular arrhythmias (coronary artery disease, previous myocardial infarction, and heart failure with maintained or reduced ejection fraction), cardiomyopathy, history of ventricular arrhythmias or cardiac arrest, chronic kidney disease (CKD), electrolytes (potassium, calcium, and magnesium), and all medication history including drugs that may increase the risk of ventricular arrhythmias as per the American Heart Association [12]. Calcium values were measured by biochemical tests and adopted values were corrected for albumin. QT prolongation was defined as values exceeding the 99th percentile (470 ms for males and 480 ms for females) [13]. Data collection was conducted and checked by HT and NT. This study was approved by the Ethics Committee of Nerima Hikarigaoka Hospital (#23122101).

Result

Among 146 cases considered as sudden death, three cases were identified where ceftriaxone and lansoprazole were used in combination. The main clinical information is presented in Table 1, and the clinical courses of the three cases are described below.

**Table 1:** Patient characteristics, clinical information, electrocardiographic findings, and laboratory tests in three cases of sudden death during concurrent use of ceftriaxone and lansoprazole.

Age (years)	Sex	Underlying conditions	Admission diagnosis	Electrocardiogram on admission	QTc interval on the electrocardiogram at admission (ms)	Latest electrolyte panels before sudden death	QT-prolonging drugs	Period of ceftriaxone usage (days)	Initial electrocardiogram at the time of sudden death
81	Male	End-stage kidney disease, paroxysmal atrial fibrillation, angina pectoris	Community-acquired pneumonia, pneumonia-associated pleural effusion	Sinus rhythm, 79 bpm, flat T waves in leads I and Ⅱ, left anterior fascicular block, prolonged QT interval	0.508	K 3.8 mmol/L, Ca 10.0 mg/dL, Mg not confirmed	Trazodone 50 mg/day	14	Pulseless electrical activity
79	Female	End-stage kidney disease, atrial flutter, myocardial infarction	Embolic cerebral infarction, aspiration pneumonia	Atrial fibrillation, 63 bpm, left posterior fascicular block, flat T waves in leads V5 and V6	0.429	K 2.8 mmol/L, Ca 9.6 mg/dL, Mg 2.3 mg/dL	None	10	Asystol
88	Female	End-stage kidney disease, hypothyroidism, hypertension, sick sinus syndrome (pacemaker implantation)	Acute pyelonephritis	Sinus rhythm, 57 bpm, complete right bundle branch block, negative T waves in leads III, and aVF, abnormal Q waves in lead aVL	0.475	K 4.6 mmol/L, Ca 9.9 mg/dL, Mg 9.9 mg/dL	None	4	Pulseless electrical activity

Case 1

**Figure 1:**
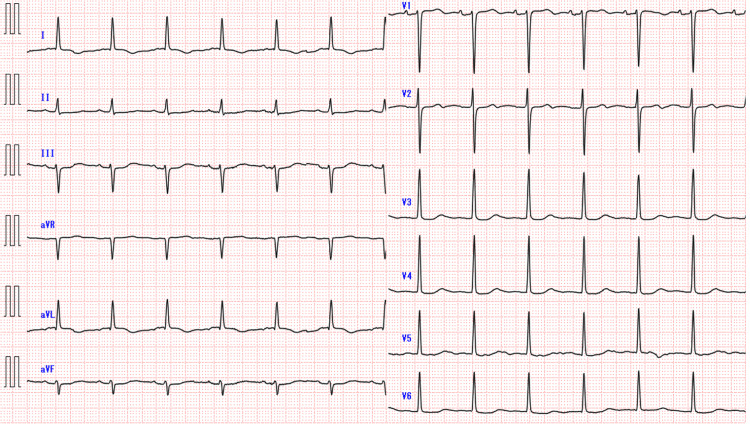
Initial electrocardiogram findings of Case 1.

An 81-year-old male with end-stage kidney disease (ESKD), paroxysmal atrial fibrillation, angina (history of percutaneous coronary intervention {PCI}), duodenal ulcer, lumbar compression fracture, and depression was on ceftriaxone (2 g/day for 14 days) for suspected pneumonia-associated pleural effusion. Upon admission, the patient's ECG revealed the following findings: sinus rhythm with a heart rate of 79 bpm, flat T waves in leads I and II, left anterior fascicular block, and a prolonged QT interval of 0.508 ms (Figure 1). He was stable with SpO_2_ at 92% (room air) until 30 min before death but was suddenly found in a state of agonal respiration with initial ECG showing pulseless electrical activity (PEA). The resuscitation code was “do not resuscitate,” and he was allowed to pass away. His admission ECG QTc was 508 ms. The last dialysis was two days before death, and on the same day, blood tests showed potassium at 3.8 mmol/L and calcium at 8.8 mg/dL with no abnormalities; magnesium was not measured (Table 2). QT-prolonging medications included 50 mg of trazodone.

**Table 2:** Laboratory results before sudden death in Case 1.

Variable	Latest data before sudden death	Reference range
Total Protein (g/dL)	6.2	6.6-8.2
Albumin (g/dL)	2.8	3.8-5.3
Urea nitrogen (mg/dL)	42.5	8-20
Creatinine (mg/dL)	5.29	0.46-0.82
Sodium (mmol/L)	129	135-145
Potassium (mmol/L)	3.8	3.6-5.0
Chloride (mmol/L)	99	98-108
Calcium (mg/dL)	8.8	8.2-10.0
Magnesium (mg/dL)	-	1.8-2.4
Glucose (mg/dL)	121	70-110

Case 2

**Figure 2:**
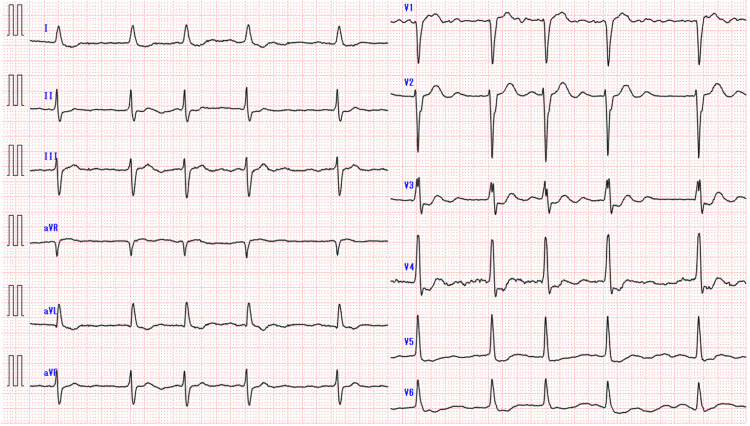
Initial electrocardiogram findings of Case 2.

A 79-year-old male with ESKD, traumatic subarachnoid hemorrhage, atrial flutter, myocardial infarction (history of PCI), and hypothyroidism was admitted for cardiogenic cerebral embolism and subsequent aspiration pneumonia. Upon admission, the ECG of Case 2 revealed the following findings: atrial fibrillation with a heart rate of 63 bpm, left posterior fascicular block, and flat T waves in leads V5 and V6 (Figure 2). He was on ceftriaxone for pneumonia for 10 days (1 g/day). Until 2 h before death, he had diarrhea but stable vital signs. He was suddenly found in cardiac arrest, with the initial ECG showing asystole. The resuscitation code was “do not resuscitate,” and he was allowed to pass away. His admission ECG showed atrial fibrillation with a QTc of 429 ms. The last dialysis was the day before death, and blood tests on the same day showed potassium at 2.8 mmol/L, corrected calcium at 9.6 mg/dL, and magnesium at 2.3 mg/dL, indicating hypokalemia (Table 3).

**Table 3:** Laboratory results before sudden death in Case 2.

Variable	Latest data before sudden death	Reference range
Total protein (g/dL)	4.6	6.6-8.2
Albumin (g/dL)	2.1	3.8-5.3
Urea nitrogen (mg/dL)	71.6	8-20
Creatinine (mg/dL)	7.4	0.46-0.82
Sodium (mmol/L)	139	135-145
Potassium (mmol/L)	2.8	3.6-5.0
Chloride (mmol/L)	98	98-108
Calcium (mg/dL)	7.7	8.2-10.0
Magnesium (mg/dL)	2.3	1.8-2.4
Glucose (mg/dL)	191	70-110

Case 3

**Figure 3:**
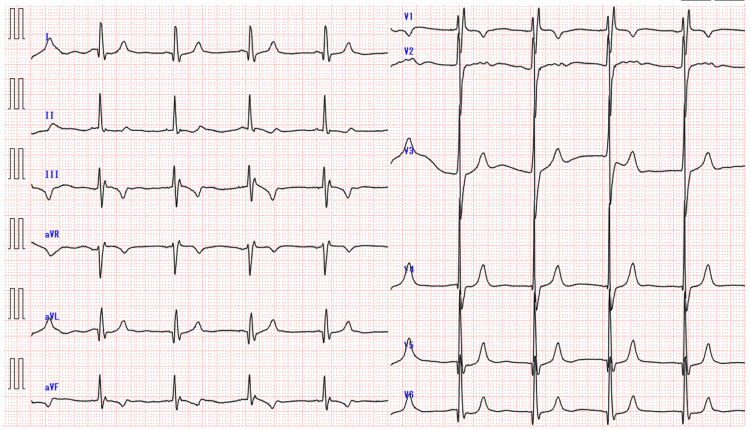
Initial electrocardiogram findings of Case 3.

An 88-year-old female with ESKD on maintenance dialysis, hypothyroidism, hypertension, and sick sinus syndrome (with a pacemaker installed) was admitted for pyelonephritis and was on ceftriaxone (1 g/day) for four days. She had increased sputum, fever (38.3℃), and decreased oxygenation (SpO_2_ 92%, nasal 2 L/min) 2 h before death, but was suddenly found in cardiac arrest with the initial ECG showing PEA. The resuscitation code was “do not resuscitate,” and she was allowed to pass away. Upon admission, the ECG of Case 3 revealed the following findings: sinus rhythm with a heart rate of 57 bpm, complete right bundle branch block, negative T waves in leads III and aVF, and abnormal Q waves in lead aVL (Figure 3). The last dialysis was two days before death, and blood tests three days before death showed potassium at 4.6 mmol/L, corrected calcium at 8.9 mg/dL, and magnesium at 2.0 mg/dL with no significant electrolyte abnormalities (Table 4).

**Table 4:** Laboratory results before sudden death in Case 3.

Variable	Latest data before sudden death	Reference range
Total protein (g/dL)	5.5	6.6-8.2
Albumin (g/dL)	3.0	3.8-5.3
Urea nitrogen (mg/dL)	37.7	8-20
Creatinine (mg/dL)	8.6	0.46-0.82
Sodium (mmol/L)	138	135-145
Potassium (mmol/L)	4.6	3.6-5.0
Chloride (mmol/L)	104	98-108
Calcium (mg/dL)	8.9	8.2-10.0
Magnesium (mg/dL)	2.0	1.8-2.4
Glucose (mg/dL)	79	70-110

Among these three cases, two had ischemic heart disease with a history of PCI, and all three were on maintenance dialysis for ESKD. Sudden death occurred on days four, 10, and 14 after starting ceftriaxone administration. One patient exhibited QT interval prolongation at the time of admission and was further administered trazodone, which exacerbates QT prolongation, during the co-administration of ceftriaxone and lansoprazole. Of the remaining two cases, one exhibited hypokalemia in a blood test performed prior to sudden death, and the other was a female patient with a QTc interval of 0.475 ms, which is at the borderline of QT prolongation.

## Discussion

Among the 146 cases considered to be sudden deaths, three occurred while the patients were on a combination of ceftriaxone and lansoprazole. In the analysis of these three cases, only one case exhibited QT prolongation on the electrocardiogram upon admission. In this case, a history of trazodone administration was confirmed. Moreover, among the remaining two cases, one showed hypokalemia in blood tests conducted before sudden death, and the other was a female patient with a borderline QT prolongation with a QTc of 0.475. Magnesium levels were measured in two cases, and none had hypomagnesemia. All cases were patients with ESKD undergoing maintenance dialysis. Bai et al. showed no significant difference in the prevalence of CKD between patients taking ceftriaxone with or without lansoprazole, and it was not a significant factor in either univariate or multivariate analysis [8]. Due to the design of this study, it is difficult to verify the causal relationship between all three cases occurring in ESKD patients. Although ceftriaxone does not require dose adjustment for renal function, some reports suggest that ceftriaxone clearance correlates with renal function [14]. In a study by Yamada et al. on ceftriaxone encephalopathy using a database of adverse drug events, risk factors identified included administration for more than 14 days, chronic kidney disease (CKD), female gender, and high dosage [15]. Trazodone, administered to the patient in Case 3 also induces QT prolongation by inhibiting the hERG channel currents in a concentration-dependent manner [16]. Therefore, it is necessary to investigate whether QT prolongation induced by the combination of ceftriaxone and lansoprazole is more likely to occur in patients with ceftriaxone accumulation, ESKD, or those taking trazodone.

There are some limitations in this study. First is the design of the study itself. This study did not investigate the causal relationship between sudden cardiac death and the combined use of ceftriaxone and lansoprazole. Instead, it aimed to elucidate the clinical progression of sudden death by leveraging the hypotheses and findings from multicenter retrospective studies by Lorberbaum et al. and Bai et al., which suggest that this combination may prolong the QT interval and elevate the risk of sudden cardiac death [6,9]. The risk of sudden death due to this combination has only recently been proposed, and further verification is desirable as the initial ECGs in this study showed asystole or PEA, and no ventricular arrhythmias, including ventricular fibrillation, were confirmed. However, if sudden death cannot be predicted from the ECG at admission, clinicians might need to avoid the combination. The risk of sudden death associated with this combination has only recently been proposed. However, this case series suggests that it may not be possible to predict sudden death from ECGs at admission, which means clinicians might need to avoid this combination. Due to the retrospective nature of the chart review and the fact that some patients were stable and not ECG monitored, we were unable to obtain ECG recordings prior to the transition to cardiac arrest. Despite establishing strict criteria for sudden death to maintain the validity of the study, the initial ECGs obtained were of cardiac arrest or PEA, with no observed instances of ventricular fibrillation or torsades de pointes, suggesting the need for further verification. The third limitation is the difference in the frequency of lansoprazole use, which is believed to vary somewhat by country, region, and medical facility. In Japan, where this study was conducted, lansoprazole is the only PPI available as an orally disintegrating tablet, making it the second most used PPI due to its ease of use in older adults [17,18]. Conversely, it is less used in Europe, and according to Bai et al., lansoprazole accounted for only 12% of all PPIs in Canada [6].

## Conclusions

In conclusion, the three cases of sudden death under the combination of ceftriaxone and lansoprazole each had an underlying factor that could cause QT prolongation. In two cases, QT prolongation was not observed at the time of admission, and it might not be predictable from the pre-medication ECG, suggesting that clinicians should avoid this drug combination until the characteristics of this potentially lethal adverse event are clearly understood.

## References

[1] van der Meer JW, Gyssens IC (2001). Quality of antimicrobial drug prescription in hospital. Clin Microbiol Infect.

[2] Pinto Pereira LM, Phillips M, Ramlal H, Teemul K, Prabhakar P (2004). Third generation cephalosporin use in a tertiary hospital in Port of Spain, Trinidad: need for an antibiotic policy. BMC Infect Dis.

[3] Fridkin SK, Steward CD, Edwards JR (1999). Surveillance of antimicrobial use and antimicrobial resistance in United States hospitals: project ICARE phase 2. Project Intensive Care Antimicrobial Resistance Epidemiology (ICARE) hospitals. Clin Infect Dis.

[4] Hayes KN, Nakhla NR, Tadrous M (2019). Further evidence to monitor long-term proton pump inhibitor use. JAMA Netw Open.

[5] Rotman SR, Bishop TF (2013). Proton pump inhibitor use in the U.S. ambulatory setting, 2002-2009. PLoS One.

[6] Bai AD, Wilkinson A, Almufleh A, Rai M, Razak F, Verma AA, Srivastava S (2023). Ceftriaxone and the risk of ventricular arrhythmia, cardiac arrest, and death among patients receiving lansoprazole. JAMA Netw Open.

[7] Shanika LG, Reynolds A, Pattison S, Braund R (2023). Proton pump inhibitor use: systematic review of global trends and practices. Eur J Clin Pharmacol.

[8] Mitsuboshi S, Imai S, Kizaki H, Hori S (2024). Concomitant use of lansoprazole and ceftriaxone is associated with an increased risk of ventricular arrhythmias and cardiac arrest in a large Japanese hospital database. J Infect.

[9] Lorberbaum T, Sampson KJ, Chang JB, Iyer V, Woosley RL, Kass RS, Tatonetti NP (2016). Coupling data mining and laboratory experiments to discover drug interactions causing QT prolongation. J Am Coll Cardiol.

[10] Andersen LW, Holmberg MJ, Berg KM, Donnino MW, Granfeldt A (2019). In-hospital cardiac arrest: a review. JAMA.

[11] Nichols L, Chew B (2012). Causes of sudden unexpected death of adult hospital patients. J Hosp Med.

[12] Tisdale JE, Chung MK, Campbell KB, Hammadah M, Joglar JA, Leclerc J, Rajagopalan B (2020). Drug-induced arrhythmias: a scientific statement from the American Heart Association. Circulation.

[13] Drew BJ, Ackerman MJ, Funk M (2010). Prevention of torsade de pointes in hospital settings: a scientific statement from the American Heart Association and the American College of Cardiology Foundation. Circulation.

[14] Simon N, Dussol B, Sampol E (2006). Population pharmacokinetics of ceftriaxone and pharmacodynamic considerations in haemodialysed patients. Clin Pharmacokinet.

[15] Yamada T, Mitsuboshi S, Suzuki K, Nishihara M, Neo M (2022). Analysis of the frequency of ceftriaxone-induced encephalopathy using the Japanese Adverse Drug Event Report database. Int J Clin Pharm.

[16] Tarantino P, Appleton N, Lansdell K (2005). Effect of trazodone on hERG channel current and QT-interval. Eur J Pharmacol.

[17] Yamamichi N, Shimamoto T, Takahashi Y, Takahashi M, Takeuchi C, Wada R, Fujishiro M (2022). Trends in proton pump inhibitor use, reflux esophagitis, and various upper gastrointestinal symptoms from 2010 to 2019 in Japan. PLoS One.

[18] Umeno J, Esaki M, Nuki Y, Kim H, Kitazono T, Matsumoto T (2013). Letter: lansoprazole consumption is more common in Japanese patients with collagenous colitis. Aliment Pharmacol Ther.

